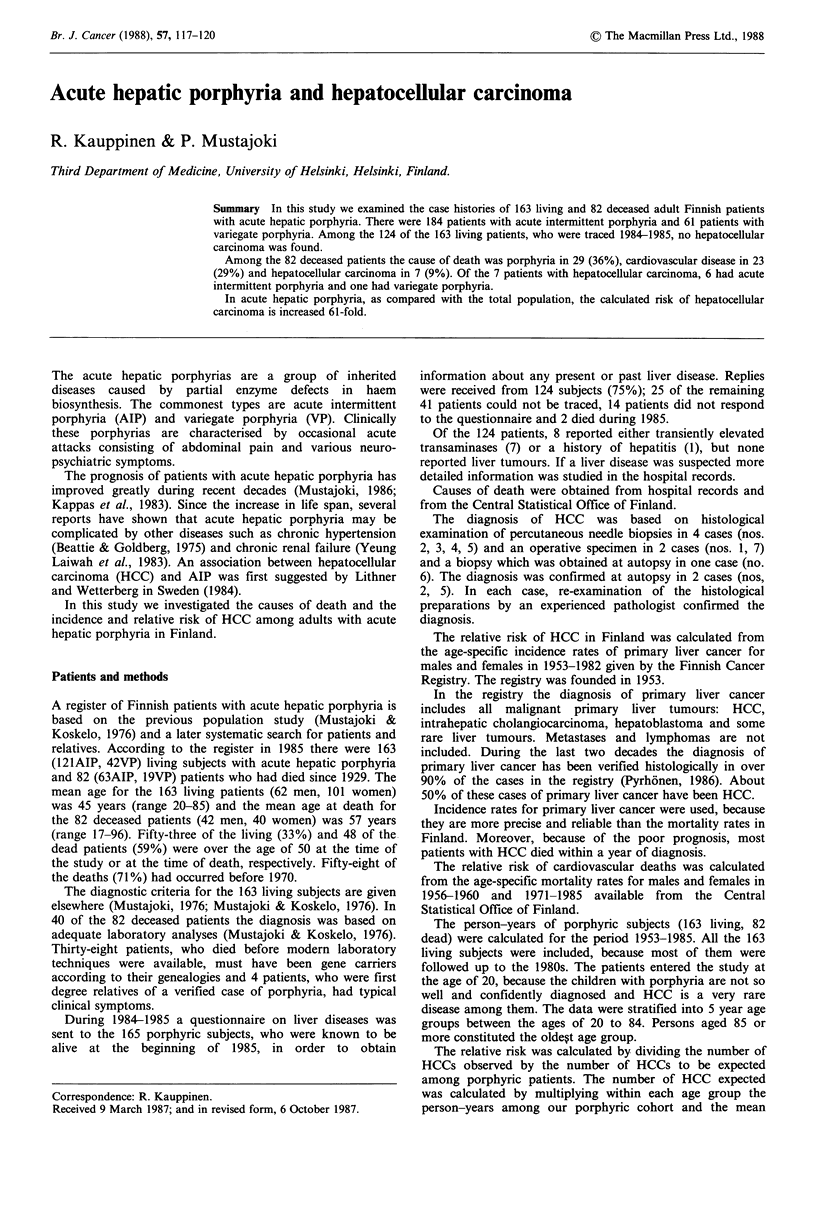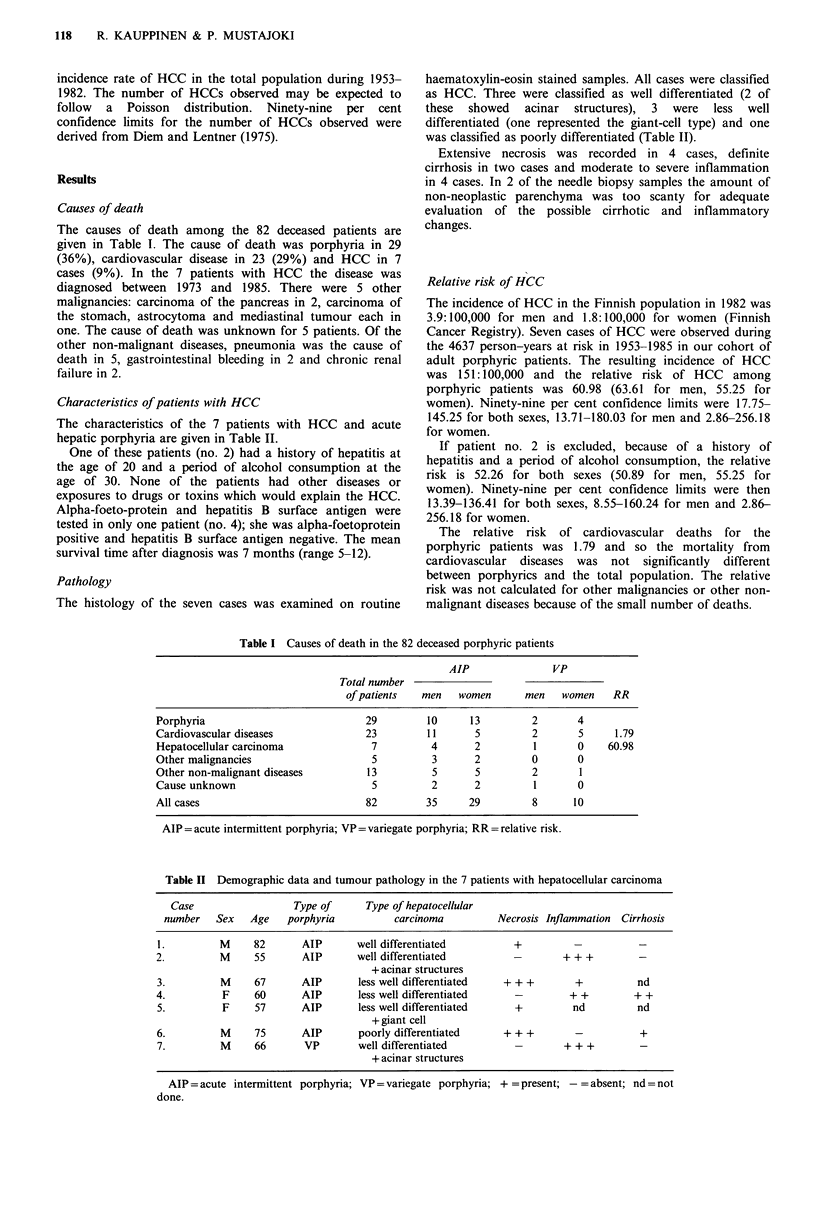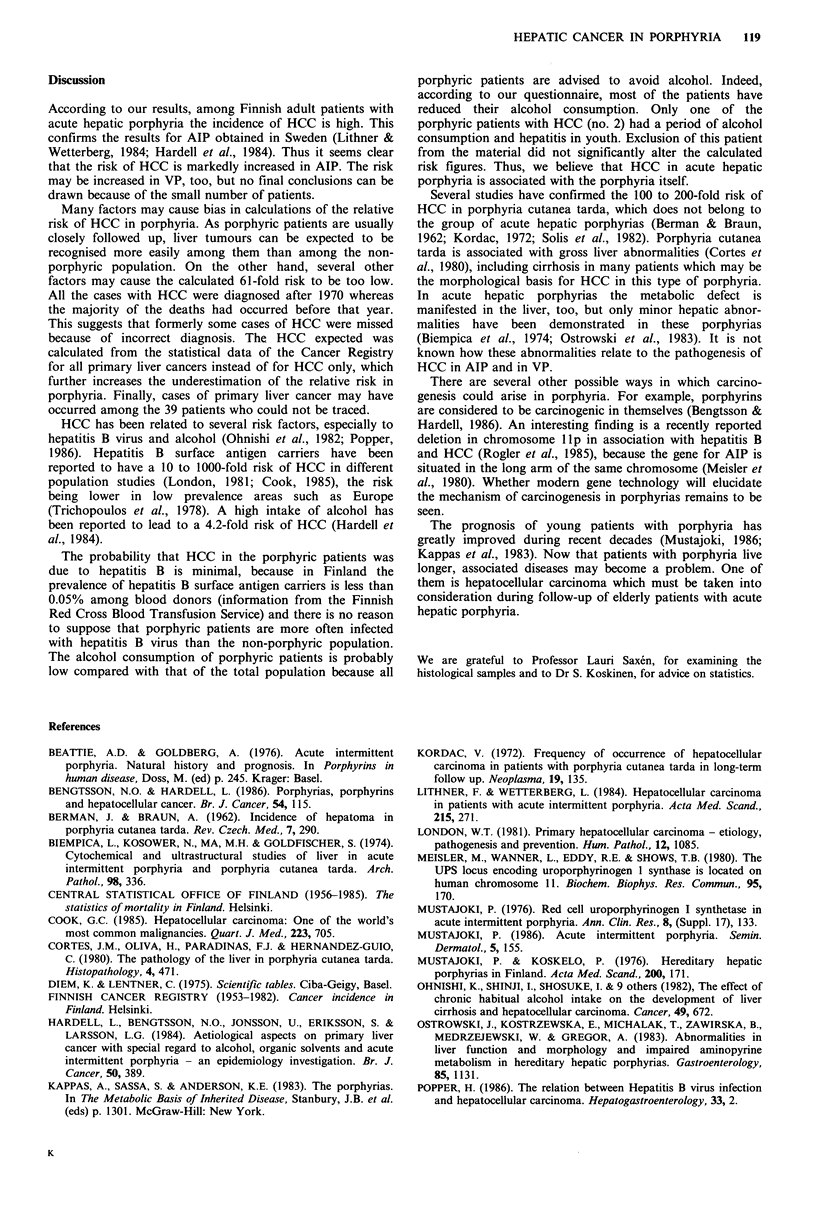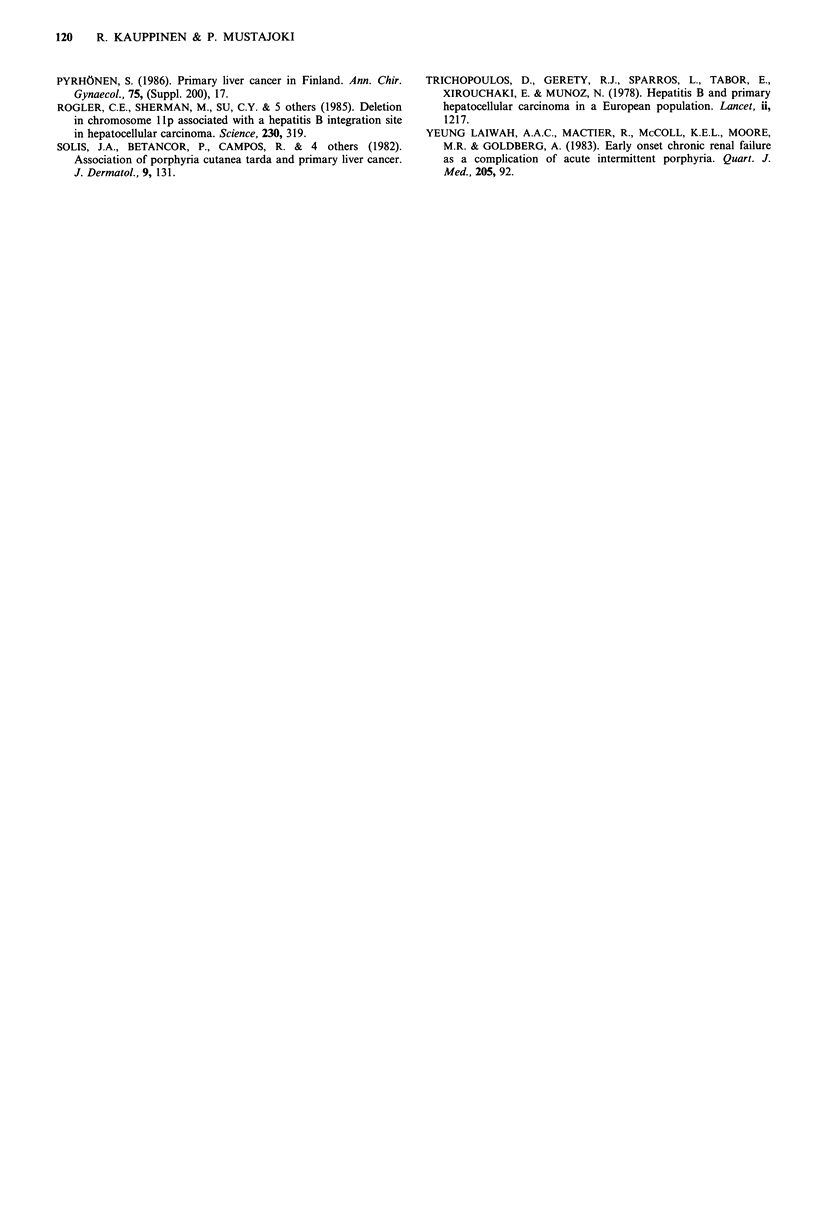# Acute hepatic porphyria and hepatocellular carcinoma.

**DOI:** 10.1038/bjc.1988.23

**Published:** 1988-01

**Authors:** R. Kauppinen, P. Mustajoki

**Affiliations:** Third Department of Medicine, University of Helsinki, Finland.

## Abstract

In this study we examined the case histories of 163 living and 82 deceased adult Finnish patients with acute hepatic porphyria. There were 184 patients with acute intermittent porphyria and 61 patients with variegate porphyria. Among the 124 of the 163 living patients, who were traced 1984-1985, no hepatocellular carcinoma was found. Among the 82 deceased patients the cause of death was porphyria in 29 (36%), cardiovascular disease in 23 (29%) and hepatocellular carcinoma in 7 (9%). Of the 7 patients with hepatocellular carcinoma, 6 had acute intermittent porphyria and one had variegate porphyria. In acute hepatic porphyria, as compared with the total population, the calculated risk of hepatocellular carcinoma is increased 61-fold.


Br. J. Cancer (1988), 57, 117-120 © The Macmillan Press Ltd., 1988

### Acute hepatic porphyria and hepatocellular carcinoma

R. Kauppinen & P. Mustajoki

*Third Department of Medicine, University of Helsinki, Helsinki, Finland.*

**Summary** In this study we examined the case histories of 163 living and 82 deceased adult Finnish patients with acute hepatic porphyria. There were 184 patients with acute intermittent porphyria and 61 patients with variegate porphyria. Among the 124 of the 163 living patients, who were traced 1984–1985, no hepatocellular carcinoma was found.

Among the 82 deceased patients the cause of death was porphyria in 29 (36%), cardiovascular disease in 23 (29%) and hepatocellular carcinoma in 7 (9%). Of the 7 patients with hepatocellular carcinoma, 6 had acute intermittent porphyria and one had variegate porphyria.

In acute hepatic porphyria, as compared with the total population, the calculated risk of hepatocellular carcinoma is increased 61-fold.

The acute hepatic porphyrias are a group of inherited diseases caused by partial enzyme defects in haem biosynthesis. The commonest types are acute intermittent porphyria (AIP) and variegate porphyria (VP). Clinically these porphyrias are characterised by occasional acute attacks consisting of abdominal pain and various neuropsychiatric symptoms.

The prognosis of patients with acute hepatic porphyria has improved greatly during recent decades (Mustajoki, 1986; Kappas *et al.*, 1983). Since the increase in life span, several reports have shown that acute hepatic porphyria may be complicated by other diseases such as chronic hypertension (Beattie & Goldberg, 1975) and chronic renal failure (Yeung Laiwah *et al.*, 1983). An association between hepatocellular carcinoma (HCC) and AIP was first suggested by Lithner and Wetterberg in Sweden (1984).

In this study we investigated the causes of death and the incidence and relative risk of HCC among adults with acute hepatic porphyria in Finland.

### Patients and methods

A register of Finnish patients with acute hepatic porphyria is based on the previous population study (Mustajoki & Koskelo, 1976) and a later systematic search for patients and relatives. According to the register in 1985 there were 163 (121AIP, 42VP) living subjects with acute hepatic porphyria and 82 (63AIP, 19VP) patients who had died since 1929. The mean age for the 163 living patients (62 men, 101 women) was 45 years (range 20–85) and the mean age at death for the 82 deceased patients (42 men, 40 women) was 57 years (range 17–96). Fifty-three of the living (33%) and 48 of the dead patients (59%) were over the age of 50 at the time of the study or at the time of death, respectively. Fifty-eight of the deaths (71%) had occurred before 1970.

The diagnostic criteria for the 163 living subjects are given elsewhere (Mustajoki, 1976; Mustajoki & Koskelo, 1976). In 40 of the 82 deceased patients the diagnosis was based on adequate laboratory analyses (Mustajoki & Koskelo, 1976). Thirty-eight patients, who died before modern laboratory techniques were available, must have been gene carriers according to their genealogies and 4 patients, who were first degree relatives of a verified case of porphyria, had typical clinical symptoms.

During 1984–1985 a questionnaire on liver diseases was sent to the 165 porphyric subjects, who were known to be alive at the beginning of 1985, in order to obtain information about any present or past liver disease. Replies were received from 124 subjects (75%); 25 of the remaining 41 patients could not be traced, 14 patients did not respond to the questionnaire and 2 died during 1985.

Of the 124 patients, 8 reported either transiently elevated transaminases (7) or a history of hepatitis (1), but none reported liver tumours. If a liver disease was suspected more detailed information was studied in the hospital records.

Causes of death were obtained from hospital records and from the Central Statistical Office of Finland.

The diagnosis of HCC was based on histological examination of percutaneous needle biopsies in 4 cases (nos. 2, 3, 4, 5) and an operative specimen in 2 cases (nos. 1, 7) and a biopsy which was obtained at autopsy in one case (no. 6). The diagnosis was confirmed at autopsy in 2 cases (nos, 2, 5). In each case, re-examination of the histological preparations by an experienced pathologist confirmed the diagnosis.

The relative risk of HCC in Finland was calculated from the age-specific incidence rates of primary liver cancer for males and females in 1953–1982 given by the Finnish Cancer Registry. The registry was founded in 1953.

In the registry the diagnosis of primary liver cancer includes all malignant primary liver tumours: HCC, intrahepatic cholangiocarcinoma, hepatoblastoma and some rare liver tumours. Metastases and lymphomas are not included. During the last two decades the diagnosis of primary liver cancer has been verified histologically in over 90% of the cases in the registry (Pyrhönen, 1986). About 50% of these cases of primary liver cancer have been HCC.

Incidence rates for primary liver cancer were used, because they are more precise and reliable than the mortality rates in Finland. Moreover, because of the poor prognosis, most patients with HCC died within a year of diagnosis.

The relative risk of cardiovascular deaths was calculated from the age-specific mortality rates for males and females in 1956–1960 and 1971–1985 available from the Central Statistical Office of Finland.

The person–years of porphyric subjects (163 living, 82 dead) were calculated for the period 1953–1985. All the 163 living subjects were included, because most of them were followed up to the 1980s. The patients entered the study at the age of 20, because the children with porphyria are not so well and confidently diagnosed and HCC is a very rare disease among them. The data were stratified into 5 year age groups between the ages of 20 to 84. Persons aged 85 or more constituted the oldeşt age group.

The relative risk was calculated by dividing the number of HCCs observed by the number of HCCs to be expected among porphyric patients. The number of HCC expected was calculated by multiplying within each age group the person–years among our porphyric cohort and the mean

Correspondence: R. Kauppinen.
Received 9 March 1987; and in revised form, 6 October 1987.

incidence rate of HCC in the total population during 1953–1982. The number of HCCs observed may be expected to follow a Poisson distribution. Ninety-nine per cent confidence limits for the number of HCCs observed were derived from Diem and Lentner (1975).

### Results

### *Causes of death*

The causes of death among the 82 deceased patients are given in Table I. The cause of death was porphyria in 29 (36%), cardiovascular disease in 23 (29%) and HCC in 7 cases (9%). In the 7 patients with HCC the disease was diagnosed between 1973 and 1985. There were 5 other malignancies: carcinoma of the pancreas in 2, carcinoma of the stomach, astrocytoma and mediastinal tumour each in one. The cause of death was unknown for 5 patients. Of the other non-malignant diseases, pneumonia was the cause of death in 5, gastrointestinal bleeding in 2 and chronic renal failure in 2.

### *Characteristics of patients with HCC*

The characteristics of the 7 patients with HCC and acute hepatic porphyria are given in Table II.

One of these patients (no. 2) had a history of hepatitis at the age of 20 and a period of alcohol consumption at the age of 30. None of the patients had other diseases or exposures to drugs or toxins which would explain the HCC. Alpha-foeto-protein and hepatitis B surface antigen were tested in only one patient (no. 4); she was alpha-foetoprotein positive and hepatitis B surface antigen negative. The mean survival time after diagnosis was 7 months (range 5–12).

### *Pathology*

The histology of the seven cases was examined on routine haematoxylin-eosin stained samples. All cases were classified as HCC. Three were classified as well differentiated (2 of these showed acinar structures), 3 were less well differentiated (one represented the giant-cell type) and one was classified as poorly differentiated (Table II).

Extensive necrosis was recorded in 4 cases, definite cirrhosis in two cases and moderate to severe inflammation in 4 cases. In 2 of the needle biopsy samples the amount of non-neoplastic parenchyma was too scanty for adequate evaluation of the possible cirrhotic and inflammatory changes.

### *Relative risk of HCC*

The incidence of HCC in the Finnish population in 1982 was 3.9:100,000 for men and 1.8:100,000 for women (Finnish Cancer Registry). Seven cases of HCC were observed during the 4637 person–years at risk in 1953–1985 in our cohort of adult porphyric patients. The resulting incidence of HCC was 151:100,000 and the relative risk of HCC among porphyric patients was 60.98 (63.61 for men, 55.25 for women). Ninety-nine per cent confidence limits were 17.75–145.25 for both sexes, 13.71–180.03 for men and 2.86–256.18 for women.

If patient no. 2 is excluded, because of a history of hepatitis and a period of alcohol consumption, the relative risk is 52.26 for both sexes (50.89 for men, 55.25 for women). Ninety-nine per cent confidence limits were then 13.39–136.41 for both sexes, 8.55–160.24 for men and 2.86–256.18 for women.

The relative risk of cardiovascular deaths for the porphyric patients was 1.79 and so the mortality from cardiovascular diseases was not significantly different between porphyrics and the total population. The relative risk was not calculated for other malignancies or other non-malignant diseases because of the small number of deaths.

**Table I** Causes of death in the 82 deceased porphyric patients

| | *Total number of patients* | *AIP* | | *VP* | | *RR* |
|---|---|---|---|---|---|---|
| | | *men* | *women* | *men* | *women* | |
| Porphyria | 29 | 10 | 13 | 2 | 4 | |
| Cardiovascular diseases | 23 | 11 | 5 | 2 | 5 | 1.79 |
| Hepatocellular carcinoma | 7 | 4 | 2 | 1 | 0 | 60.98 |
| Other malignancies | 5 | 3 | 2 | 0 | 0 | |
| Other non-malignant diseases | 13 | 5 | 5 | 2 | 1 | |
| Cause unknown | 5 | 2 | 2 | 1 | 0 | |
| All cases | 82 | 35 | 29 | 8 | 10 | |

AIP = acute intermittent porphyria; VP = variegate porphyria; RR = relative risk.

**Table II** Demographic data and tumour pathology in the 7 patients with hepatocellular carcinoma

| *Case number* | *Sex* | *Age* | *Type of porphyria* | *Type of hepatocellular carcinoma* | *Necrosis* | *Inflammation* | *Cirrhosis* |
|---|---|---|---|---|---|---|---|
| 1. | M | 82 | AIP | well differentiated | + | – | – |
| 2. | M | 55 | AIP | well differentiated + acinar structures | – | + + + | – |
| 3. | M | 67 | AIP | less well differentiated | + + + | + | nd |
| 4. | F | 60 | AIP | less well differentiated | – | + + | + + |
| 5. | F | 57 | AIP | less well differentiated + giant cell | + | nd | nd |
| 6. | M | 75 | AIP | poorly differentiated | + + + | – | + |
| 7. | M | 66 | VP | well differentiated + acinar structures | – | + + + | – |

AIP = acute intermittent porphyria; VP = variegate porphyria; + = present; – = absent; nd = not done.

### Discussion

According to our results, among Finnish adult patients with acute hepatic porphyria the incidence of HCC is high. This confirms the results for AIP obtained in Sweden (Lithner & Wetterberg, 1984; Hardell *et al*., 1984). Thus it seems clear that the risk of HCC is markedly increased in AIP. The risk may be increased in VP, too, but no final conclusions can be drawn because of the small number of patients.

Many factors may cause bias in calculations of the relative risk of HCC in porphyria. As porphyric patients are usually closely followed up, liver tumours can be expected to be recognised more easily among them than among the non-porphyric population. On the other hand, several other factors may cause the calculated 61-fold risk to be too low. All the cases with HCC were diagnosed after 1970 whereas the majority of the deaths had occurred before that year. This suggests that formerly some cases of HCC were missed because of incorrect diagnosis. The HCC expected was calculated from the statistical data of the Cancer Registry for all primary liver cancers instead of for HCC only, which further increases the underestimation of the relative risk in porphyria. Finally, cases of primary liver cancer may have occurred among the 39 patients who could not be traced.

HCC has been related to several risk factors, especially to hepatitis B virus and alcohol (Ohnishi *et al*., 1982; Popper, 1986). Hepatitis B surface antigen carriers have been reported to have a 10 to 1000-fold risk of HCC in different population studies (London, 1981; Cook, 1985), the risk being lower in low prevalence areas such as Europe (Trichopoulos *et al*., 1978). A high intake of alcohol has been reported to lead to a 4.2-fold risk of HCC (Hardell *et al*., 1984).

The probability that HCC in the porphyric patients was due to hepatitis B is minimal, because in Finland the prevalence of hepatitis B surface antigen carriers is less than 0.05% among blood donors (information from the Finnish Red Cross Blood Transfusion Service) and there is no reason to suppose that porphyric patients are more often infected with hepatitis B virus than the non-porphyric population. The alcohol consumption of porphyric patients is probably low compared with that of the total population because all porphyric patients are advised to avoid alcohol. Indeed, according to our questionnaire, most of the patients have reduced their alcohol consumption. Only one of the porphyric patients with HCC (no. 2) had a period of alcohol consumption and hepatitis in youth. Exclusion of this patient from the material did not significantly alter the calculated risk figures. Thus, we believe that HCC in acute hepatic porphyria is associated with the porphyria itself.

Several studies have confirmed the 100 to 200-fold risk of HCC in porphyria cutanea tarda, which does not belong to the group of acute hepatic porphyrias (Berman & Braun, 1962; Kordac, 1972; Solis *et al*., 1982). Porphyria cutanea tarda is associated with gross liver abnormalities (Cortes *et al*., 1980), including cirrhosis in many patients which may be the morphological basis for HCC in this type of porphyria. In acute hepatic porphyrias the metabolic defect is manifested in the liver, too, but only minor hepatic abnormalities have been demonstrated in these porphyrias (Biempica *et al*., 1974; Ostrowski *et al*., 1983). It is not known how these abnormalities relate to the pathogenesis of HCC in AIP and in VP.

There are several other possible ways in which carcinogenesis could arise in porphyria. For example, porphyrins are considered to be carcinogenic in themselves (Bengtsson & Hardell, 1986). An interesting finding is a recently reported deletion in chromosome 11p in association with hepatitis B and HCC (Rogler *et al*., 1985), because the gene for AIP is situated in the long arm of the same chromosome (Meisler *et al*., 1980). Whether modern gene technology will elucidate the mechanism of carcinogenesis in porphyrias remains to be seen.

The prognosis of young patients with porphyria has greatly improved during recent decades (Mustajoki, 1986; Kappas *et al*., 1983). Now that patients with porphyria live longer, associated diseases may become a problem. One of them is hepatocellular carcinoma which must be taken into consideration during follow-up of elderly patients with acute hepatic porphyria.

We are grateful to Professor Lauri Saxén, for examining the histological samples and to Dr S. Koskinen, for advice on statistics.

### References

BEATTIE, A.D. & GOLDBERG, A. (1976). Acute intermittent porphyria. Natural history and prognosis. In *Porphyrins in human disease*, Doss, M. (ed) p. 245. Krager: Basel.

BENGTSSON, N.O. & HARDELL, L. (1986). Porphyrias, porphyrins and hepatocellular cancer. *Br. J. Cancer*, **54**, 115.

BERMAN, J. & BRAUN, A. (1962). Incidence of hepatoma in porphyria cutanea tarda. *Rev. Czech. Med.*, **7**, 290.

BIEMPICA, L., KOSOWER, N., MA, M.H. & GOLDFISCHER, S. (1974). Cytochemical and ultrastructural studies of liver in acute intermittent porphyria and porphyria cutanea tarda. *Arch. Pathol.*, **98**, 336.

CENTRAL STATISTICAL OFFICE OF FINLAND (1956–1985). *The statistics of mortality in Finland*. Helsinki.

COOK, G.C. (1985). Hepatocellular carcinoma: One of the world's most common malignancies. *Quart. J. Med.*, **223**, 705.

CORTES, J.M., OLIVA, H., PARADINAS, F.J. & HERNANDEZ-GUIO, C. (1980). The pathology of the liver in porphyria cutanea tarda. *Histopathology*, **4**, 471.

DIEM, K. & LENTNER, C. (1975). *Scientific tables*. Ciba-Geigy, Basel.

FINNISH CANCER REGISTRY (1953–1982). *Cancer incidence in Finland*. Helsinki.

HARDELL, L., BENGTSSON, N.O., JONSSON, U., ERIKSSON, S. & LARSSON, L.G. (1984). Aetiological aspects on primary liver cancer with special regard to alcohol, organic solvents and acute intermittent porphyria – an epidemiology investigation. *Br. J. Cancer*, **50**, 389.

KAPPAS, A., SASSA, S. & ANDERSON, K.E. (1983). The porphyrias. In *The Metabolic Basis of Inherited Disease*, Stanbury, J.B. *et al.* (eds) p. 1301. McGraw-Hill: New York.

KORDAC, V. (1972). Frequency of occurrence of hepatocellular carcinoma in patients with porphyria cutanea tarda in long-term follow up. *Neoplasma*, **19**, 135.

LITHNER, F. & WETTERBERG, L. (1984). Hepatocellular carcinoma in patients with acute intermittent porphyria. *Acta Med. Scand.*, **215**, 271.

LONDON, W.T. (1981). Primary hepatocellular carcinoma – etiology, pathogenesis and prevention. *Hum. Pathol.*, **12**, 1085.

MEISLER, M., WANNER, L., EDDY, R.E. & SHOWS, T.B. (1980). The UPS locus encoding uroporphyrinogen 1 synthase is located on human chromosome 11. *Biochem. Biophys. Res. Commun.*, **95**, 170.

MUSTAJOKI, P. (1976). Red cell uroporphyrinogen I synthetase in acute intermittent porphyria. *Ann. Clin. Res.*, **8**, (Suppl. 17), 133.

MUSTAJOKI, P. (1986). Acute intermittent porphyria. *Semin. Dermatol.*, **5**, 155.

MUSTAJOKI, P. & KOSKELO, P. (1976). Hereditary hepatic porphyrias in Finland. *Acta Med. Scand.*, **200**, 171.

OHNISHI, K., SHINJI, I., SHOSUKE, I. & 9 others (1982), The effect of chronic habitual alcohol intake on the development of liver cirrhosis and hepatocellular carcinoma. *Cancer*, **49**, 672.

OSTROWSKI, J., KOSTRZEWSKA, E., MICHALAK, T., ZAWIRSKA, B., MEDRZEJEWSKI, W. & GREGOR, A. (1983). Abnormalities in liver function and morphology and impaired aminopyrine metabolism in hereditary hepatic porphyrias. *Gastroenterology*, **85**, 1131.

POPPER, H. (1986). The relation between Hepatitis B virus infection and hepatocellular carcinoma. *Hepatogastroenterology*, **33**, 2.

PYRHÖNEN, S. (1986). Primary liver cancer in Finland. *Ann. Chir. Gynaecol.*, **75**, (Suppl. 200), 17.

ROGLER, C.E., SHERMAN, M., SU, C.Y. & 5 others (1985). Deletion in chromosome 11p associated with a hepatitis B integration site in hepatocellular carcinoma. *Science*, **230**, 319.

SOLIS, J.A., BETANCOR, P., CAMPOS, R. & 4 others (1982). Association of porphyria cutanea tarda and primary liver cancer. *J. Dermatol.*, **9**, 131.

TRICHOPOULOS, D., GERETY, R.J., SPARROS, L., TABOR, E., XIROUCHAKI, E. & MUNOZ, N. (1978). Hepatitis B and primary hepatocellular carcinoma in a European population. *Lancet*, **ii**, 1217.

YEUNG LAIWAH, A.A.C., MACTIER, R., McCOLL, K.E.L., MOORE, M.R. & GOLDBERG, A. (1983). Early onset chronic renal failure as a complication of acute intermittent porphyria. *Quart. J. Med.*, **205**, 92.